# Supplementary material for: A systematic approach to identify recycling endocytic cargo depending on the GARP complex
Source: eLife. 2019 Jan 29;8:e42837. doi: 10.7554/eLife.42837 (PMC6374077; doi:10.7554/eLife.42837)
Supplement: Supplementary file 1. [file elife-42837-supp1.docx]

**Tab. 1** Used yeast strains

| **Strain** | **Genotype** | **Reference** |
| --- | --- | --- |
| FFY541 | *SEY6210 MATα leu2-3,112 ura3-52 his3-∆200 trp-∆901 lys2-801 suc2-∆9 GAL* | Florian Fröhlich |
| FFY535 | *SEY6210 MATα leu2-3,112 ura3-52 his3-∆200 trp-∆901 lys2-801 suc2-∆9 GAL URA3::pBW2406_ADHpr-OSTIR-9myc VPS53-AID-6xHA::HPH* | This study |
| FFY838 | *SEY6210 Mat α leu2-3,112 ura3-52 his3-Δ200 trp1-Δ901 suc2-Δ9 lys2-801; GAL pRS305_ADHpr-OSTIR-3xFlag::LEU VPS53-AID-6xHA::HPH* | This study |
| FFY837 | *SEY6210 MATα leu2-3,112 ura3-52 his3-Δ200 trp1-Δ901 suc2-Δ9 lys2-801; GAL pRS305_ADHpr-OSTIR-3xFlag::LEU VPH1-mCherry::KAN* | This study |
| FFY906 | *SEY6210 MATα leu2-3,112 ura3-52 his3-Δ200 trp1-Δ901 suc2-Δ9 lys2-801; GAL pRS305_ADHpr-OSTIR-3xFlag::LEU VPS53-AID-6xHA::HPH VPH1-GFP::KAN* | This study |
| FFY1034 | *SEY6210 MATα leu2-3,112 ura3-52 his3-∆200 trp-∆901 lys2-801 suc2-∆9 GAL VPS53-AID-6xHA::HPH VPH1-mcherry::KAN* | This study |
| FFY851 | *SEY6210 MATα leu2-3,112 ura3-52 his3-Δ200 trp1-Δ901 suc2-Δ9 lys2-801; GAL pRS305_ADHpr-OSTIR-3xFlag::LEU VPH1-mCherry::KAN VPS53-AID-6xHA::HPH* | This study |
| FFY925 | *SEY6210 MATα leu2-3,112 ura3-52 his3-∆200 trp-∆901 lys2-801 suc2-∆9 GAL pRS305_ADHpr-OSTIR-3xFLAG::LEU* | This study |
| FFY1008 | *SEY6210 MATα leu2-3,112 ura3-52 his3-∆200 trp-∆901 lys2-801 suc2-∆9 GAL VPS53-AID-6xHA::HPH* | This study |
| FFY943 | *SEY6210 MATα leu2-3,112 ura3-52 his3-∆200 trp-∆901 lys2-801 suc2-∆9 GAL URA3::pBW2406_ADH1pr-OSTIR1-9myc VPS53-AID-6xHA::HPH pep4∆::NAT* | This study |
| FFY944 | *SEY6210 MATα leu2-3,112 ura3-52 his3-Δ200 trp1-Δ901 suc2-Δ9 lys2-801; GAL pRS305_ADHpr-*OSTIR-3xFLAG::LEU VPH1-mCherry::KAN VPS53-AID-6xHA::HPH *pep4∆::*NAT | This study |
| FFY954 | *SEY6210 MATα leu2-3,112 ura3-52 his3-Δ200 trp1-Δ901 suc2-Δ9 lys2-801; GAL pRS305_ADHpr-OSTIR1-3xFlag::LEU VPS53-AID-6xHA::HPH VPH1-mCherry::TRP PDR12-neonGreen::KAN* | This study |
| FFY1040 | *SEY6210 MATα leu2-3,112 ura3-52 his3-Δ200 trp1-Δ901 suc2-Δ9 lys2-801; GAL pRS305_ADHpr-OSTIR1-3xFlag::LEU VPH1-mCherry::TRP PDR12-neonGreen::KAN* | This study |
| FFY1050 | *SEY6210 MATα leu2-3,112 ura3-52 his3-∆200 trp-∆901 lys2-801 suc2-∆9 GAL pRS305_ADHpr-OSTIR-3xFLAG::LEU DNF2-neonGreen::KAN VPH1-mCherry::TRP* | This study |
| FFY1015 | *SEY6210 MATα leu2-3,112 ura3-52 his3-Δ200 trp1-Δ901 suc2-Δ9 lys2-801; GAL pRS305_ADHpr-OSTIR-3xFlag::LEU VPS53-AID-6xHA::HPH VPH1-mCherry::TRP ADHpr-DNF2-neonGreen::KAN* | This study |
| FFY1143 | *SEY6210 MATα leu2-3,112 ura3-52 his3-∆200 trp-∆901 lys2-801 suc2-∆9 GAL pRS305_ADHpr-OSTIR-3xFLAG::LEU VPH1-mCherry::TRP LEM3-neonGreen::KAN* | This study |
| FFY1142 | *SEY6210 MATα leu2-3,112 ura3-52 his3-Δ200 trp1-Δ901 suc2-Δ9 lys2-801; GAL pRS305_ADHpr-OSTIR-3xFlag::LEU VPS53-AID-6xHA::HPH VPH1-mCherry::TRP LEM3-neonGreen::KAN* | This study |
| FFY1066 | *SEY6210 MATα leu2-3,112 ura3-52 his3-Δ200 trp1-Δ901 suc2-Δ9 lys2-801; GAL pRS305_ADHpr-OSTIR-3xFlag::LEU VPH1-mCherry::TRP VPS10-neonGreen::KAN* | This study |
| FFY1067 | *SEY6210 MATα leu2-3,112 ura3-52 his3-Δ200 trp1-Δ901 suc2-Δ9 lys2-801; GAL pRS305_ADHpr-OSTIR-3xFlag::LEU VPS53-AID-6xHA::HPH VPH1-mCherry::TRP VPS10-neonGreen::KAN* | This study |
| FFY607 | *SEY6210 MATα leu2-3,112 ura3-52 his3-Δ200 trp1-Δ901 ade2-101 suc2-Δ9; GAL vps53Δ::NAT* | This study |
| FFY1144 | *SEY6210 MATα leu2-3,112 ura3-52 his3-∆200 trp-∆901 lys2-801 suc2-∆9 GAL lem3Δ::NAT* | This study |
| FFY1014 | *SEY6210 Mat α leu2-3,112 ura3-52 his3-Δ200 trp1-Δ901 suc2-Δ9 lys2-801; GAL pRS305_ADHpr-OSTIR-3xFlag::LEU VPS53-6xHA::HPH* | This study |
| FFY1195 | *leu2-3,112 ura3-52 his3-Δ200 trp1-Δ901 suc2-Δ9 lys2-801; GAL pRS305_ADHpr-OSTIR-3xFLAG::LEU VPS53-AID-6xHA::HPH TEFpr-LEM::NAT* | This study |
| FFY1281 | *leu2-3,112 ura3-52 his3-∆200 trp-∆901 lys2-801 suc2-∆9 GAL pRS305_ADHpr-OSTIR-3xFLAG::LEU TEFpr-LEM::NAT* | This study |
| FFY1208 | *W303 MATa leu2-3,112 trp1-1 ura3-1 his3-11,15 pho8∆::HIS3 pho13∆::TRP* | Schuck et al., JCS 2014 |
| FFY1209 | *W303 MATa leu2-3,112 trp1-1 ura3-1 his3-11,15 pho8∆::HIS3 pho13∆::TRP pRS305_ADHpr-OSTIR-3xFLAG::LEU VPS53-AID-6xHA::HPH DNF2-PHO8Δ60::KAN* | This study |
| FFY1210 | *W303 MATa leu2-3,112 trp1-1 ura3-1 his3-11,15 pho8∆::HIS3 pho13∆::TRP pRS305_ADHpr-OSTIR-3xFLAG::LEU VPS53-AID-6xHA::HPH LEM3-PHO8Δ60::KAN* | This study |
| FFY1211 | *W303 MATa leu2-3,112 trp1-1 ura3-1 his3-11,15 pho8∆::HIS3 pho13∆::TRP pRS305_ADHpr-OSTIR-3xFLAG::LEU VPS53-AID-6xHA::HPH ITR1-PHO8Δ60::KAN* | This study |
| FFY1212 | *W303 MATa leu2-3,112 trp1-1 ura3-1 his3-11,15 pho8∆::HIS3 pho13∆::TRP pRS305_ADHpr-OSTIR-3xFLAG::LEU VPS53-AID-6xHA::HPH TAT1-PHO8Δ60::KAN* | This study |
| FFY1213 | *W303 MATa leu2-3,112 trp1-1 ura3-1 his3-11,15 pho8∆::HIS3 pho13∆::TRP pRS305_ADHpr-OSTIR-3xFLAG::LEU VPS53-AID-6xHA::HPH PDR12-PHO8Δ60::KAN* | This study |
| FFY1299 | *SEY6210 Mat α leu2-3,112 ura3-52 his3-Δ200 trp1-Δ901 suc2-Δ9 lys2-801; GAL pRS305_ADHpr-OSTIR-3xFlag::LEU VPS53-AID-6xHA::HPH apl1Δ* | This study |
| FFY1305 | *SEY6210 MATα leu2-3,112 ura3-52 his3-Δ200 trp1-Δ901 suc2-Δ9 lys2-801; GAL pRS305_ADHpr-OSTIR-3xFlag::LEU VPS53-AID-6xHA::HPH VPH1-mCherry::TRP LEM3-neonGreen::KAN apl1∆::NAT* | This study |
| FFY1524 | *SEY6210 MATα leu2-3,112 ura3-52 his3-Δ200 trp1-Δ901 suc2-Δ9 lys2-801; GAL pRS305_ADHpr-OSTIR-3xFlag::LEU VPS53-AID-6xHA::HPH VPH1-mCherry::TRP lem3*Δ::NAT | This study |
