# Supplementary material for: A systematic approach to identify recycling endocytic cargo depending on the GARP complex
Source: eLife. 2019 Jan 29;8:e42837. doi: 10.7554/eLife.42837 (PMC6374077; doi:10.7554/eLife.42837)
Supplement: Supplementary file 2. [file elife-42837-supp2.docx]

**Tab. 2** Used plasmids

| **plasmid** | **Reference** |
| --- | --- |
| pRS305_ADHpr_OsTir_3xFlag | Robbie Loewith |
| pFA6a_Pho8Δ60_kanMX 6 | Sebastian Schuck, J. Cell Sci. (2014) |
